# Economic Recovery in the Age of COVID-19

**DOI:** 10.1007/s10272-020-0929-6

**Published:** 2020-12-01

**Authors:** Robert Skidelsky

**Affiliations:** Millbank House, 1 Millbank, SW1P 3JU London, UK

## Abstract

With the world on the brink of yet another steep recession, and with ecological disaster looming, we can no longer afford the luxury of an economic policy which concentrates on the fight against inflation, leaves unemployment to emergency measures, distribution of wealth and income to the market, and ignores ecological challenges.

The COVID-19 pandemic is an invitation to what the economist Joseph Schumpeter called creative destruction: a chance to liquidate obsolete investments and to create something new, better, and, in the jargon, more 'resilient' and 'sustainable'. Schumpeter understood that humankind does not progress in a balanced way, rather it lurches from one extreme to another, each extreme producing its own reaction.

In political economy, the subject of this contribution, the excesses of the Keynesian social democracy in the 1970s brought about the extreme reaction of neo-liberalism. The hubris of neo-liberalism – its failure to guard against the ever present possibility of collapse, its inattention to social justice, its reckless embrace of globalisation, its Faustian pact with consumerism – has in turn bred a reaction, but to what is as yet unclear. Populist forces of Right and Left, made up of fragments of old and new discontents, compete for the succession. The balance remains elusive.

We have both a short-term and long-term crisis on our hands. In the short term we run the risk of what some analysts are calling the Third Great World Depression. In the long run the risk is of the exhaustion of Nature's tolerance for our profligate habits. The prize is to enfold our pandemic recovery measures into a long-term strategy for a sustainable way of life – I will not say growth, for growth as we understand it may not be sustainable.

The short-term threat to jobs and livelihoods is clear enough. Much of Europe is on a life-support system. The world economy will have shrunk by about 5% in 2020, and hopes of a V-shaped recovery have been put on hold. This means that unemployment is set to go on rising through 2021. In the UK, we expect an unemployment rate of close to 10%. Everyone is waiting for the end of the coronavirus pandemic so that we can get back to 'normal'. But the virus is no longer the main problem: it is the scarring of the economy produced by a prolonged and continuous period of non-work which will damage the recovery.

* This article is based on Robert Skidelsky's keynote speech at the Intereconomics/CEPS online conference "COVID-19: From Lockdown to Recovery", 27 October 2020.

**Robert Skidelsky**, Professor emeritus, University of Warwick, Coventry, UK.

And what about the further future? Suppose by a heroic effort we succeed in reopening economies much as they were before. Can anyone say they were in a healthy or sustainable situation before the pandemic struck? Debt-driven growth models which produced consumption and asset booms followed by financial busts: that *was* the pre-pandemic normal.

I am often asked, what would British economist John Maynard Keynes have said? I will try to give my answer, though I hasten to add that Keynes, from beyond the grave, has not authorised me to do so. Nor is Keynes the last word on the matters I will deal with.

## Then and now

My book *Keynes: The Return of the Master* (Skidelsky, 2009) was published in the autumn of 2009. It was published the year after the global banking collapse of 2008 and the massive rescue operations undertaken by governments all round the world – not just the bailout of a bankrupt banking system, but also large monetary and fiscal stimulus. This activism was in contrast to the 'do nothing' stance of governments following the Wall Street crash of 1929. I believe that it prevented another Great Depression: the fall in output following the 2008 collapse was limited to four quarters, whereas output went on falling for thirteen quarters after the collapse of 1929.

Long before recovery was secure, however, our own stimulus was terminated. Alarmed by the deficits they had incurred, governments started to slash public spending. The 'return of the master' proved brief. "I guess everyone is a Keynesian in a foxhole" said Robert Lucas, high priest of Chicago economics. Keynes was for emergencies only.

And we have the same reaction today. Compelled to close down a large fraction of their economies to stop the spread of the coronavirus contagion, governments have spent money freely to keep up the incomes of millions of people prevented from working. But they continue to hope that as the economy reopens, a V-shaped recovery will relieve them of their fiscal burden. The talk is of 'fiscal sustainability', the need for consolidation and debt reduction even as the economy is set to shrink.

Many would see this as a reasonable position. Most economists view the market system as fundamentally healthy. It will get sick from time to time and therefore need medication, but it is basically self-healing, like the human body. So treatment should be limited in scope and duration. This is particularly the case given the unreliability of political medicine. Keynes rejected this analogy between the market system and the self-healing body and believed rather that a market system left unattended by the state could never be healthy.

### Keynes for beginners

Keynes's revolutionary insight was that capitalist market economies do not have an automatic tendency towards full employment. This assertion shocked the economists of Keynes's day, whose models taught them that persisting unemployment was impossible if wages were flexible. Keynes's Cambridge colleague Arthur Pigou expressed the typical belief of 1933: "With perfectly free competition...there will always be a strong tendency for wage-rates to be so related to demand that everyone is employed" (Pigou, 1933). Based on this argument, unemployed workers must be *choosing* not to work. Seeing the millions unemployed all around him during the Great Depression, Keynes thought: There is something wrong with your model! These people are not *choosing* not to work. They cannot *find* work at any wage.

Keynesian economics starts with this blinding shaft of common sense. People are unemployed when there is no *demand* for their services. Yet this insight never fully converted the economics profession, who went on cooking up all kinds of fancy reasons to demonstrate that what looked like unwanted unemployment was really a 'choice for leisure'. Today I would wager that most economists believe, deep down, that most unemployed people could find work if they really wanted to, or if state benefits did not provide them with an alternative income.

But why do economies not quickly bounce back from collapses? Surely, if an employer does not want to employ me at £500 a week, I can always lower my wage requirement till it becomes profitable for him to employ me. By insisting on unrealistic wages, workers are 'pricing themselves out of employment'. But Keynes gave two reasons why even flexible wages will not maintain or restore full employment.

#### Wages and demand

Keynes's first argument was that every producer is also a consumer: my wages are your income, because my wages buy your goods. If my wages go down, your income goes down, too. The general principle is that cuts in production costs (whether by cutting wages or by laying off workers) deepen a slump by simultaneously cutting total demand or spending power. A fall of income in one part of the economy reduces production in another part, and so on, in a downward spiral as unemployment spreads rapidly throughout the economy. Eventually spending power is stabilised at a much lower level as people stop saving. But nothing has happened to stimulate consumption, and therefore to promote a recovery. The weird idea that the way to revive an economy is by getting everyone to stop spending comes only to a well-trained neo-classical economist.

#### Confidence and money

Keynes's second argument against the V-shaped recovery model had to do with the behaviour of money. It is characteristic of a slump that instead of investing their money, businesses 'hoard' it, or 'add to their cash reserves'. The greater this 'liquidity preference' is, the higher the rate of interest that owners of money will charge to lend it out. But to stimulate production, borrowers need lower rates, not higher rates. So when confidence is low, the higher rates demanded by the banks for loans mean even less investment, less consumption and less employment.

In this way, flexibility of wages and stickiness of interest rates combine to deepen the slump. Contrary to Robert Lucas, without government 'stimulus' the economy will remain stuck in the foxhole.

But the mainstream economist has a comeback: depressions or deep recessions are very rare events, like Nassim Taleb's 'black swans' (2008). So it is absurd to organise economic life as if the next slump is just around the corner. Market economies have built-in stability so powerful that slumps will be very rare. This is exactly what Keynes denied: black swans can fly out of a clear blue sky at any time. We have just seen a large flock of them in 2020.

The reason, Keynes said, was that the theory of the 'self-equilibrating' market economy depends on the idea that everyone, and particularly investors, can accurately predict the future. If they can accurately calculate what assets they buy today will be worth in ten years time, they would never buy things at the wrong prices. As Keynes (1937) wrote: "The calculus of probability...was supposed to be capable of reducing uncertainty to the same calculable status as certainty itself". But this was a myth. "Actually...we have as a rule, only the vaguest idea of any but the most direct consequences of our acts" (Keynes, 1936). This was the second huge shaft of commonsense to pierce the mathematical precision of forecasting models.

And a huge consequence followed. Because the future is uncertain, private investment – which depends on the expectation of future yield – will be unsteady. Prosperity will depend on peoples' 'animal spirits'. When they are feeling confident, they hire more workers; when they are pessimistic, they hire fewer.

## Stabilisation policy

Two conclusions follow from this account of market behaviour: 1) Collapses are always possible because the future is uncertain; and 2) When they happen, there are no 'automatic' market mechanisms to ensure a quick bounce back.

That is why governments are indispensable 'balancers' of market economies. They add and subtract spending power as and when needed.

This explains why there is no virtue in trying to balance the budget as such. Being Keynesian means having a theory of the economy that justifies the use of the state budget to balance economic activity at an optimal level of output and employment. This can mean either a budget surplus or a budget deficit or a balanced budget, depending on what is happening in the economy. It is the accounts of the economy that it needs to balance. Without this balancing act the economy will have a spontaneous tendency not to full employment but to underemployment.

To maintain economic life on a balanced, even keel, governments need to do two things.

First, they need to steady the rate of investment. They can do this through public investment programmes. Keynes (1936) wrote: "I expect to see the state, which is in a position to [take] long views...taking an ever greater responsibility for directly organising investment". This happened in the 25 years after World War II, but since the 1980s, the state's share in total investment has fallen drastically, increasing economic instability. Notice that a large state investment share is not just a policy for the foxhole but a permanent part of economic management.

Secondly, governments should pursue counter-cyclical policy to limit the effect of remaining fluctuations. This means injecting extra spending into the economy when private spending falls and curtailing it when it rises. It can be done on the tax side, or spending side, or both. The 'multiplier', based on what is called 'the marginal propensity to consume', tells governments what the multiplied effect of any spending they add to or subtract from the economy will be.

The answer to the failures of both old-fashioned Keynesianism and newfangled monetarism is not to abandon the balancing role of the state, but to make it as automatic as possible. The state should commit to two things: a rolling programme of public investment and a public sector job guarantee.

The first would reduce fluctuations in investment to much narrower limits; the second would provide a buffer stock of jobs, which would automatically expand in a downturn and deplete in an upturn.

Public investment does not require public ownership. Much of it could be done by quasi-state institutions like public investment banks or funds or state-holding companies. These would operate under a broad central government mandate or 'mission' to use Mariana Mazzucato's word, that reflects national purpose, but which insulates commercial decisions from political meddling.

The 'counter-cyclical' public sector job programme would be centrally financed, but with projects chosen and administered locally. The result of both policies pursued together would be to abolish unwanted unemployment for the first time since the Industrial Revolution.

These two balancing functions, public investment and counter-cyclical policy, are needed to ensure the full employment and stability of capitalist market economies. And the fuller the use of a country's human resources, the more prosperous the country will be, the greater the social contentment and the less the danger of political extremism. This – in a nutshell – is the message of Keynes for our day.

## Three objections

Now let us consider three neoclassical objections to Keynesian theory and policy.

First, as we have seen, economists believe the market economy to be much more naturally stable than Keynes supposed. Hence they view Keynesian demand management as inherently destabilising. History does not support them. The most stable period in modern times, with the fullest employment and fastest growth rate, has been the period from 1950 to1975, when Keynesian theory and policy was in control.

Second, anti-Keynesian economists teach that public investment 'crowds out' private investment. This is true if a government adds to public spending when all the economy's resources are already fully employed. It is a cousin of the idea that public borrowing merely adds to the burdens of future generations. But whenever there is spare capacity, public investment can 'crowd in' private investment by increasing total demand for goods and services. Most governments drastically cut public investment after the 1970s. Growth was halved and unemployment rose. Some public investment is bound to be 'wasted', but this has to be compared with the waste of unemployment.

Third, monetarist economists – descendants of Milton Friedman – claim that Keynesian counter-cyclical policy is inherently inflationary. Vote-catching will lead Keynesian politicians to print too much money, resulting in creeping and eventually accelerating inflation.

Behind the monetarist argument is the belief that there is only one shock against which policy has to guard: government, which is governments 'monkeying around' with money. Instability in the price level can delude people into trading at false prices, disturbing the natural equilibrium of market transactions. If the key to economic stability is a low and constant rate of inflation, then control of the money supply (or equivalently) of interest rates needs to be taken out of the hands of politicians and vested in independent central banks.

History gives only qualified support to the monetarist thesis. Inflation was subdued throughout the 25 years of the Keynesian era and only started to rise at the end of the 1960s, for reasons much more connected with the Vietnam War than with Keynesian economics. As for the inherent stability of an economy with stable money: a decade of low inflation did not prevent the financial collapse of 2008-09.

Nor did quantitative easing – flooding the economy with central bank 'base money' or M0 in 2009-12 – bring about a robust recovery after the collapse. The aim of quantitative easing was to lower the cost of borrowing by forcing up the price of bonds. Its fallacy lay in the belief that the 'money supply' (which includes 'broad money' or bank credit) is directly under the control of the central bank. How much bank credit an expansion of base money brings about depends on Keynes's 'animal spirits'. A very high rate of interest can sometimes kill off a boom; but even a negative rate of interest might not produce recovery if expectations of profit from increased investment are zero.

Neoclassical economists are not the only critics of Keynesianism. Marxists would claim that such an updated Keynesian programme is just pie in the sky. A capitalist economy needs a 'reserve army of the unemployed' to keep up profits by keeping down wages. Only a fully socialised economy, they say, can abolish unemployment and maintain wage growth. The answer is that between 1950 and 1975, Keynesian-managed capitalist economies averaged unemployment rates of 2% to 3%, half of what they have been since; they had doubled the growth rates we have since had, with rising rather than stagnating wages; and at a cost in inflation only slightly higher than we have experienced under monetarist management.

No system of political economy is perfect. But it should be judged not by comparison with some ideal system, but with the realistic alternatives. Keynes set out to save democracy from the two challengers of his day – fascism and communism. He said that if we continued with laissez-faire in the face of massive unemployment, political freedom would not survive. "But it may be possible by a right analysis of the problem to cure the disease whilst preserving efficiency and freedom" (Keynes, 1936).

I would echo him today. I doubt if western populations will for much longer tolerate a political economy that delivers persisting underemployment, frequent crashes, stagnant wages, and extreme inequalities of wealth and income.

In thinking about our post-COVID-19 world, Keynes is an excellent start, but he did not solve all economic problems. Although he assumed that the desire to consume more would eventually be satiated by abundance, he had no inkling of long-run ecological constraints on growth. Keynes understood that inequality was both an economic and ethical problem, but his theoretical work was directed to overcoming unemployment, the big problem of the day, and he did not link it to the unequal distribution of wealth, which we are much more likely to do today.

## European context

Let me conclude by putting what I have just said into a European context. This is a serious question, because while the rules of the European Union prevent member states from pursuing Keynesian policies at the national level, there is no provision for European-wide Keynesianism.

The fundamental design flaw of the eurozone has often been pointed out; it created a monetary union without three crucial tools which are needed to stabilise economies: a budget big enough to act as a balancer, a fiscal transfer capacity to deal with asymmetric shocks and a lender of last resort for the banking system. These were not accidental omissions. The European Economic and

Monetary Union was built on the belief that they were unnecessary. The creators of the Union accepted the Friedman monetary doctrine that rule-governed market economies are naturally stable, which was consistent with the long-standing anti-inflationary views of the German Bundesbank. But it was worse than that: the eurozone treaty forbade the use of stabilising tools at the national level. What this meant is that the Union as a whole was badly equipped to deal with the kinds of shocks to which market capitalism is prone.

The conventional, or German, view of the financial crisis of 2008-09 was that it resulted from excessive public debts and fiscal profligacy of the Mediterranean countries. Had these countries balanced their budgets as the rules prescribed, the financial shock could have been avoided. The alternative, and I think correct, view, is that the Union provided no non-deflationary mechanism for adjusting current account imbalances between its members. Keynes's remark of 1941 applies very accurately to the EU: "The process of adjustment is compulsory for the debtor, and voluntary for the creditor: the debtor must borrow; the creditor is under no such compulsion." His Clearing Union set out to remedy this design flaw in the global system by providing for creditor adjustment, but no such mechanism was established in Europe. As result, Germany in particular was left free to pile up current account surpluses without limit. The system was maintained by an unstable system of creditor loans which dried up the moment debtors got into trouble.

With the fiscal policy of all member states constrained by balanced buget rules and public debt ceilings, monetary policy – the weaker stabilisation instrument – was the only macro policy available. Mario Draghi, European Central Bank (ECB) president, found a way of bending the rules of the ECB sufficiently to rescue the EU from collapse in 2015. But he recognised the limitations of a purely monetary stimulus. In an interview with *Financial Times*, Draghi (2019) said:

> I [have] talked about fiscal policy as a necessary complement to monetary policy since 2014. Now the need is more urgent than before. Monetary policy will continue to do its job but the negative side effects as you more forward are more and more visible. Monetary pumping worked, but more feebly that fiscal pumping would have.

Draghi proposed a budget for the eurozone large enough to be a stabilising tool: this has not been acted on.

The COVID-19 crisis has brought one promising institutional innovation. In July of this year, the European Commission proposed a €750 billion European Recovery Fund, dubbed Next Generatioan EU. This would authorise the Commission to borrow in the capital markets on the EU's behalf and disburse the loans raised as grants and loans, split half-and-half to its members.

However, it has two obvious limitations. The European Council called the fund 'an exceptional response to temporary but extreme circumstances'. In other words, it is not intended to become a permanent part of the EU's institutional structure, so it is limited in size, scope and duration.

And secondly, it still has to be agreed by all 27 member states. As a result of continuous wrangling, no budgetary allocations have yet been made. What seemed like an imaginative leap forward looks like a quagmire. As one analyst has remarked "a sword of Damocles therefore hangs over the whole plan".

Many member states are still betting on that V-shaped recovery. Germany and France are planning to cut their deficits next year (Germany from 6.35% to 4.25%) with the 'peak of the stimulus' seemingly past. The calls for consolidation are like those leading to self-induced double dip of 2011.

## Conclusion

With the world on the brink of yet another steep recession, and with ecological disaster looming, we can no longer afford the luxury of an economic policy which concentrates on the fight against inflation, leaves unemployment to emergency measures, distribution of wealth and income to the market, and ignores ecological challenges.

Overcoming the scourage of unemployment, connecting its treatment to issues of just distribution nationally and globally, and linking both to a Green New Deal: this tripartite task is the biggest politico-economic challenge facing us.
